# The relationship between depressiveness and eating behaviors among women

**DOI:** 10.1192/j.eurpsy.2025.1405

**Published:** 2025-08-26

**Authors:** K. Rachubińska, D. Schneider-Matyka, E. Grochans, I. Cerzniewska, A. M. Cybulska

**Affiliations:** 1 Department of Nursing, Pomeranian Medical University, Szczecin, Poland

## Abstract

**Introduction:**

Research has demonstrated the effect of eating disorders on the occurrence of depressiveness as well as the effect of depressiveness on the occurrence of eating disorders.

**Objectives:**

The objective of the study was to determine the relationship between depressiveness and the occurrence of eating disorders, i.e., emotional eating, uncontrolled eating, cognitive restraint of eating, and the risk of orthorexia.

**Methods:**

The study was conducted among 556 women from the West Pomeranian Voivodeship (Poland). The study employed the diagnostic survey method using a questionnaire technique: The Beck Depression Inventory, the ORTO—15 Questionnaire, the Three-Factor Eating Questionnaire, and a sociodemographic questionnaire.

**Results:**

Higher depressiveness severity is associated with a higher score on the “Cognitive Restraint of Eating” scale. The authors’ original study demonstrated a statistically significant relationship only between depressiveness and the “Uncontrolled Eating” subscale (*p* = 0.001).

**Table 1.** A multivariate model without moderation—analysis of the effect of sociodemographic variables and the severity of depressiveness according to the BDI on cognitive restraint of eating according to TFEQ-13

**Table:** 

	Level	ß	−95% CI	+95% CI	t	*p*
Marital status	Single (ref.)					
	In a relationship	0.091	0.005	0.176	2.084	0.038
Professional activity	Inactive (ref.)					
	Active	−0.046	−0.135	0.043	−1.008	0.314
Age		−0.166	−0.260	−0.073	−3.511	<0.001
BDI (scoring)		0.228	0.147	0.309	5.527	<0.001

ref.—reference level, ß—standardized regression coefficient, CI—confidence interval, and BDI—Beck Depression Inventory

**Table 2.** A multivariate model with moderation—analysis of the effect of sociodemographic variables and the severity of depressiveness according to the BDI on cognitive restraint of eating according to TFEQ-13

**Table:** 

	ß	−95% CI	+95% CI	t	*p*
Absolute term				1.914	0.056
Marital status٭BDI	0.015	−0.147	0.176	0.178	0.859
Age٭BDI	−1.344	−6.233	3.545	−0.540	0.589
Professional activity٭BDI	−0.037	−0.236	0.162	−0.362	0.717
Educational background٭BDI	0.013	−0.145	0.171	0.165	0.869
Residence ٭BDI	0.153	0.002	0.305	1.994	0.047

ref.—reference level, ß—standardized regression coefficient, CI—confidence interval, BDI—Beck Depression Inventory, and ٭ moderation effect

**Conclusions:**

The results of this study suggest that depressiveness is an important factor that contributes to a better understanding of eating behaviors. In addition, the results of this study suggest that eating behaviors and psychological factors should be taken into account in psychological interventions in the treatment of eating disorders. The clinical goal can be considered to be an improvement in non-normative eating behaviors, such as a reduction in overeating episodes or eating less frequently in the absence of a feeling of hunger.

**Disclosure of Interest:**

None Declared

